# Uptake and regulation of resource allocation for optimal plant performance and adaptation to stress

**DOI:** 10.3389/fpls.2013.00455

**Published:** 2013-11-14

**Authors:** Yong-Ling Ruan, John W. Patrick, Sergey Shabala, Thomas L. Slewinski

**Affiliations:** ^1^School of Environmental and Life Sciences, The University of NewcastleCallaghan, NSW, Australia; ^2^Australia-China Research Centre for Crop Improvement, The University of NewcastleCallaghan, NSW, Australia; ^3^School of Agricultural Science, University of TasmaniaHobart, TAS, Australia; ^4^Department of Plant Biology, Cornell UniversityIthaca, NY, USA

**Keywords:** resource allocation, adaptation to stress, plant stress responses, crop yield, food security

Resource allocation is fundamental to plant development, yield formation and tolerance to abiotic and biotic stress. Resources comprise both organic and inorganic forms. The former includes organic carbon (C) such as sugars; whereas the latter covers mineral ions and water. In most plants, organic C is initially produced in photosynthetic leaves as sucrose that is transported through phloem to non-photosynthetic tissues (sinks) for diverse uses. On the other hand, ions and water are taken up by roots from soil and transported to the aerial parts through xylem. It has taken mankind thousands of years to breed plant varieties that allocate resources into readily harvestable organs that are compatible with high intensity agricultural systems. However, most of the changes in the resource partitioning, imposed by plant breeding, conflict with the evolutionary trajectories of the particular species. Thus, further modifying resource allocation in plants remains challenging. At the same time, food production will need to increase by up to 70% by 2050 to match population growth (e.g., Godfray et al., 2010). Achieving this goal has become increasingly challenging. For instance, availability of arable land is progressively decreasing as a result of urbanization. This is further aggravated by global climate change driving increased frequency and severity of drought, flooding and heat stress events in many regions of the world (Gilbert, 2009; Godfray et al., 2010). Thus, it is imperative to advance our fundamental understanding of the mechanisms underpinning resource uptake and allocation in plants from molecular to whole plant levels. With this background, we believe it is timely to launch this research topic on uptake and regulation of resource allocation. The 14 papers published in the topic highlight some major advances and future directions in the area.

Improving photosynthetic efficiency is a key strategy to increase plant biomass production. In this context, Slewinski (2013) discussed novel ways to engineer kranz-type C4 photosynthesis based on a new hypothesis that describes how the syndrome can repeatedly and rapidly evolve from conserved developmental programs found in C3 angiosperms. Membrane transport between intracellular compartments and cell types play significant roles in mediating flows of photosynthetic products (photoassimilates) from chloroplasts of source leaves to sites of utilization within sinks. To this end, Ludewig and Flügge (2013) provide an insightful update on the expanding array of discovered membrane transporters that contribute to these photoassimilate flows. Apart from efflux to cytosol for synthesis of sucrose, some fixed C in chloroplasts is also used for starch synthesis at daytime that is degraded into glucose and maltose for sucrose synthesis and export at night. Here, Hirose et al. (2013) identified a rice α-glucan water dikinase as a key enzyme for starch degradation, a mutation of which caused hyper-accumulation of starch in source leaves without detectable effects on vegetative growth whilst significantly impeding reproductive development. Rapid homeostatic adjustments of source to sink photoassimilate flows likely depend upon post-translational control of transporter activities. Krügel and Kuehn (2013) review this emerging area of exciting research endeavor with a focus on protein-protein interactions regulating activities of sucrose/H^+^ symporters (SUT). Mapping SUT expression profiles throughout the source-path-sink system has provided an information base for Milne et al. (2013) to propose potential roles that SUTs may play in accumulating sucrose to high concentrations in Sorghum stems. Understanding mechanisms responsible for determining partitioning of photoassimilates (and other resources) between competing sinks continues to remain elusive. Against the current knowledge of phloem transport biology, Patrick (2013) re-evaluates Don Fisher's high-pressure manifold hypothesis in which plasmodesmata located in the sink phloem are conceived to exert a primary control over photoassimilate partitioning patterns.

Upon reaching sinks, assimilates are unloaded from phloem for sink development and yield formation. To this end, Bihmidine et al. (2013) provide an update on the major molecular drivers of sink strength covering the potential roles of recently identified SWEET sugar uniporters and novel aspects of a sucrose-degrading enzyme, invertase (INV). On a related note, new pathways mediated by INV during maize endosperm development have been explored by Silva-Sanchez et al. (2013) by comparing protein profiles between the INV-mutant (minature1) and its wild-type. Carbon derived from photosynthetically-generated sucrose is used for the synthesis of a diverse range of storage products including cellulose (e.g., Brill et al., 2011), starch (e.g., Ruan et al., 2008) and fructan. Here, Van den Ende (2013) propose two emerging roles for fructans and raffinose family oligosaccharides (RFOs): (i) their contribution to overall cellular reactive oxygen species (ROS) homeostasis by specific ROS scavenging processes and (ii) their action as phloem-mobile signaling molecules under stress. Apart from their nutrient roles, soluble sugars, including sucrose, glucose and fructose and their derivatives such as trehalose and trehalose-6-phosphate, function as signals regulating gene expression and cell development. The sugars may function as independent signals or through crosstalk with other signaling pathways as highlighted by Wang and Ruan (2013) on the regulation of cell division and expansion by sugar and auxin signaling.

A plant's ability to assimilate carbon and allocate carbohydrates is critically dependent on potassium availability (Cakmak, 2005). In this volume, Sharma et al. (2013) provide a comprehensive overview of molecular mechanisms behind K+ transport and sequestration in the model plant *Arabidopsis thaliana*, and their regulation under diverse stress conditions. Potassium also is a major inorganic osmolyte responsible for cell turgor maintenance and all types of plant movements, from stomata movement to tropisms and tissue expansion growth (Shabala, 2003). As a result, K+ transport in plants is causally related to flow of water between various tissues and cellular compartments. The latter issue is addressed by Prado and Maurel (2013) in which they evaluate the regulation of leaf hydraulics from molecular to whole plant levels emphasizing the role of aquaporins in this process. They also discuss how the above knowledge could help in optimizing plant performance and its adaptation to extreme conditions over short- and long-time scales.

Resource allocation is also central to plant defense responses to abiotic and biotic stresses. The tug of war between plants and their pathogens for resources is highlighted by Schultz et al. (2013). These authors describe the discovery of how plants, as a major defense strategy, mitigate pathogen attack by altering their patterns of resource allocation. In relation to abiotic stress, Liu et al. (2013) assess current understanding of sugar regulation of fruit and seed responses to heat and drought from which they formulate new directions for improving seed and fruit set, which are key determinants of crop yield potential (Ruan et al., 2012).

Overall, it is becoming increasingly clear that uptake and regulation of resource allocation is a rapidly advancing field, with ever increasing significance for sustainable agriculture. We hope this research topic will shed new light on some major aspects of resource allocation and boost research in this area of critical importance for future food security and environmental sustainability.
